# Incidence and Risk Factors of Cholestasis in Newborns with Hemolytic Disease—A Case-Control Study

**DOI:** 10.3390/jcm13113190

**Published:** 2024-05-29

**Authors:** Agnieszka Drozdowska-Szymczak, Natalia Mazanowska, Tomasz Pomianek, Artur Ludwin, Paweł Krajewski

**Affiliations:** 1 Department of Neonatology and Neonatal Intensive Care, Institute of Mother and Child, Kasprzaka 17a, 01-211 Warsaw, Poland; agnieszka.drozdowska@imid.med.pl (A.D.-S.); tomasz.pomianek@imid.med.pl (T.P.); pawel.krajewski@imid.med.pl (P.K.); 2 Department of Obstetrics and Gynecology, Institute of Mother and Child, Kasprzaka 17a, 01-211 Warsaw, Poland; 3 Department of Obstetrics and Gynecology, Medical University of Warsaw, Pl. Starynkiewicza 1/3, 02-015 Warsaw, Poland; ludwin@cm-uj.krakow.pl

**Keywords:** newborn, cholestasis, hemolytic disease of the newborn, HDN, IUT, HDFN, direct bilirubin

## Abstract

**Background/Objectives**: One of the rare causes of cholestasis may be hemolytic disease of the fetus and newborn (HDFN). **Methods**: We retrospectively analyzed 88 medical records of HDFN newborns with cholestasis and 186 records of children with HDFN without cholestasis and conducted an observational, case-control, retrospective study. **Results**: Factors influencing the risk of cholestasis were lower gestational age at birth (36.83 ± 1.9 vs. 37.57 ± 1.8, *p* = 0.002), Rh or Kidd HDFN (80.7% vs. 53.2%), and the need for intrauterine transfusion (27.3 vs. 11.8%). The subjects had lower hemoglobin concentrations at birth (14.01 ± 3.8 vs. 16.39 ± 2.8 g/dL) and during whole hospital stay, higher cord blood total bilirubin concentration (4.26 ± 1.8 vs. 2.39 ± 1.4 mg/dL), higher maximum bilirubin concentration (15.27 ± 5.8 vs. 10.24 ± 3.4 mg/dL), and more frequent liver ultrasound abnormalities (19.9 vs. 6.3%). They also required more extended hospitalization due to higher rates of postnatal blood transfusion (33 vs. 3.8%), more frequent need for exchange transfusion (8.8% vs. 2.2%), more extended time and higher risk of phototherapy (94.3 vs. 59.1%), and higher usage of immunoglobulins (55.7 vs. 8.1%), parenteral nutrition (45.5 vs. 12.9%), and antibiotics (14.8 vs. 4.8%). **Conclusions**: The risk factors for cholestasis in children with HDFN are lower gestational age at delivery, Rh and Kidd serological type of HDFN, and the need for intrauterine transfusions.

## 1. Introduction

Cholestasis refers to decreased bile flow due to obstruction of bile outflow tracts or lower secretion by hepatocytes due to impaired liver function. This results in elevated levels of direct bilirubin, bile acids, and other bile components. According to new guidelines, the diagnosis can be made if the direct bilirubin level in serum exceeds 1 mg/dL (17.1 µmmol/L), regardless of the total bilirubin concentration [1,2,3]. Clinical manifestations of cholestasis may include jaundice (varying in severity), olive skin discoloration, pale stools, and hepatomegaly [2]. Cholestasis can be divided into extrahepatic, when the cause is obstructed bile outflow, or intrahepatic, when the cause is liver cell damage or intrahepatic bile duct hypoplasia.

Both free and direct bilirubin concentrations may be increased in patients with hemolytic disease of the fetus and newborn (HDFN). Cholestasis in these children is caused by liver overload with stored iron. It happens more often in children requiring intrauterine blood transfusions, usually with anti-D immunization [4]. It could also be caused by liver overload by excessive amounts of conjugated bilirubin. Bilirubin is formed from the breakdown of heme, a component of hemoglobin. Due to its poor water solubility, bilirubin in plasma (free or indirect bilirubin) is bound with albumin and can cross the blood-brain barrier with potential neurotoxic effects [5,6]. Free bilirubin is transported to the liver and transformed into water-soluble direct bilirubin that cannot penetrate the blood-brain barrier [6,7] Direct bilirubin is secreted into the bile and enters the intestine, where bacterial enzymes convert it into bile pigments—urobilinogen [6,7].

In fetomaternal immunization, when fetal erythrocytes pass through the placenta, the mother can produce specific antibodies against antigens inherited from the father. Those can also pass through the placenta and coat fetal erythrocytes, leading to hemolysis [5,6,7,8]. Released bilirubin poses no danger to the fetus because it is disposed of through the placenta [8]. The situation differs after birth because the newborn’s immature liver enzymatic system cannot cope with the excessive bilirubin. It may lead to kernicterus and, consequentially, even cerebral palsy [5,7,8]. The increase in bilirubin concentration can be rapid, so every child diagnosed with hemolytic disease of the fetus and newborn (HDFN) is closely monitored and, if necessary, treated with intensive phototherapy [5,8]. In resistant cases, exchange transfusion may be required, with a risk of serious complications such as thrombocytopenia, hypocalcemia, hypernatremia, hypomagnesemia, hyperkalemia, hypoglycemia, leukopenia, sepsis, embolism, thrombosis, necrotizing enterocolitis, cardiac arrhythmias, and even death [5,9,10,11,12]. Some authors have described using immunoglobulin infusions to treat hyperbilirubinemia [5,12,13], including the latest AAP guidelines from 2022 [14]. However, a 2018 Cochrane analysis found insufficient data to recommend such a management [15]. 

This study aimed to analyze the incidence and risk factors for cholestasis in newborns from pregnancies complicated with hemolytic disease of the newborn.

## 2. Materials and Methods

### 2.1. Study Design

This is a single-center retrospective observational case-control study. The approval was issued by the Institutional Review Board (Bioethics Committee of the Medical University of Warsaw ethical approval No: AKBE/260/2022 issued on the 7 November 2022). 

The STROBE protocol was used to report the study [16].

### 2.2. Setting

The study included all the HDFN newborns admitted between 1 January 2017 and 31 December 2020 to the Neonatology Ward of the First Department of Obstetrics and Gynecology, Medical University of Warsaw. At that time, this tertiary center was locally centralizing care on pregnancies complicated by fetomaternal immunization.

### 2.3. Participants

The complete hospital records of newborns with HDFN complicated with cholestasis were retrospectively compared with HDFN newborns without cholestasis. The study compared possible exposure factors between the groups.

### 2.4. Variables

We assessed the incidence of variable factors in the study group (newborns with cholestasis and HDFN) and the control group (HDFN newborns with normal bilirubin levels). During statistical analysis, we considered parameters such as sex, gestational age at delivery, prematurity, birth weight, hypotrophy, serologic types of HDFN, the need for intrauterine transfusions, and liver ultrasound results. The laboratory parameter assessment included direct bilirubin serum concentration, total bilirubin serum concentration at birth and maximal value during hospitalization, hemoglobin concentration at birth and the nadir during hospitalization, ferritin concentration, TSH level, and urine test results for CMV PCR. The therapeutic procedures analyzed included phototherapy, intravenous immunoglobulin or antibiotic therapy, intravenous fluids (10% glucose infusion or parenteral nutrition), deferral of enteral nutrition, and the need for exchange transfusion in children in each group. We also compared the length of hospital stay. 

### 2.5. Data Sources/Measurement

The data extracted from hospital records were analyzed. According to new guidelines, cholestasis was diagnosed when the direct bilirubin concentration was above 1 mg/dL [1,2,3].

All the patients with known fetomaternal immunization were followed up with serial ultrasound examinations during pregnancy. The indication for intrauterine transfusion was an MoM value exceeding 1.5 for the peak systolic flow velocity (PSV) in the fetal middle cerebral artery (MCA). Experienced maternal-fetal medicine specialists performed the procedures according to a predetermined protocol, with the volume of transfused blood calculation based on measured and expected fetal hemoglobin levels [17,18].

After delivery, a cord blood sample was taken in every newborn with known fetomaternal immunization to measure the total bilirubin concentration and complete blood count. In patients diagnosed after delivery, laboratory tests from venous blood were made directly after the diagnosis. Consequently, in children born from pregnancies with prenatally diagnosed hemolytic disease, the bilirubin concentration was usually measured on the first day of life; in children with HDFN in the ABO groups, it was usually the following days of life. 

All patients had direct bilirubin measured at least once. The ferritin levels were checked in newborns with severe hemolysis or after intrauterine transfusions. TSH was measured in a subgroup of children during the differential diagnosis of cholestasis—14 out of 88. CMV was measured when the direct bilirubin concentration increased in subsequent tests. Directly after drawing the blood sample, all the samples were analyzed by highly qualified laboratory diagnosticians in a hospital’s central lab. 

In the first week of life, an abdominal ultrasound was performed. The examination was performed by a radiologist or neonatologist certified to perform ultrasound examinations. 

Phototherapy and exchange transfusion were performed per the American Academy of Pediatrics recommendations for children born at ≥35 weeks of pregnancy and the recommendations developed by Vinod K. Butani et al. for children born <35 weeks of pregnancy [11,13,19]. In high-risk patients (e.g., after intrauterine transfusions or with a rapid increase in bilirubin concentration), phototherapy was initiated directly after the delivery. Immunoglobulin infusion was usually administered when the total bilirubin concentration was 2–3 mg/dL lower than the threshold value for exchange transfusion. In newborns qualified for exchange transfusion, IVIG (IntraVenous ImmunoGlobulin) was also started during preparation for the procedure. In some cases, the exchange transfusion was subsequently withheld due to a marked reduction in bilirubin concentration.

### 2.6. Bias

The selection bias might lead to a higher incidence of cholestasis in our study due to the more severe cases of HDFN treated in our center. Patients with a severe course of fetomaternal immunization were referred for intrauterine therapy. Therefore, newborns with milder disease might have been delivered in local hospitals.

### 2.7. Statistical Methods

Categorical variables are presented as numbers of subjects and percentages. The variables that showed normal distribution were presented as mean ± standard deviation, and other continuous variables were non-normally distributed and presented as median values with lower and upper quartiles. The Kolmogorov–Smirnov test verified normal distribution. The minimum and maximum values of all continuous variables were presented. Continuous data were compared using student *t*-tests or the Mann–Whitney U-test, depending on the distribution. Categorical data were compared using the chi-square test and Fisher’s exact test. A *p*-value of less than 0.05 was considered statistically significant. Data were analyzed using SPSS software v.21 (IBM Corp., Armonk, NY, USA).

Only records with no missing data were considered, and there was no loss to follow-up because the whole hospital stay was in our center. 

## 3. Results

Between the 1st of January 2017 and the 31st of December 2020 in the First Department of Obstetrics and Gynecology, Medical University of Warsaw, 88 children with cholestasis in the course of HDFN were born (study group). The control group consisted of 186 children with HDFN who did not develop cholestasis. The incidence of cholestasis in HDFN patients treated in our center was 32.1% 

A comparison was made between children with elevated direct bilirubin concentration (study group; *n* = 88; 32.1%) and those with direct bilirubin within a normal range (controls, *n* = 186; 67.9%). The results are shown in Table 1, Table 2 and Table 3. The characteristics of both study and control groups differed only when gestational age at delivery was considered (36.83 ± 1.9 and 37.57 ± 1.8, *p* = 0.002, respectively), other parameters such as birth weight, the prevalence of small-for-gestation babies and gender were comparable between both groups. Cholestasis was more frequent in children born prematurely (*p* = 0.002). Children with cholestasis had more often hemolytic disease caused by the presence of Rh (*p* < 0.001) or Kidd antibodies (*p* = 0.014), and less frequently by ABO incompatibility (*p* = 0.001), and no differences were found in the presence of other serological types of antibodies (Kell, Duffy, MNS) or when there was more than one serological type of antibody present. We confirmed a higher risk of cholestasis in children treated with intrauterine transfusions (*p* 11.8% vs. 27.3%, *p* = 0.001). Further details can be found in Table 1.

The mean direct bilirubin concentration at the timepoint of the first measurement was 0.63 ± 0.6 mg/dL in the studied cases, with the maximum concentration during hospital stay reaching 2.56 ± 6.2 mg/dL. In two children, extreme values were observed (50.2 mg/dL and 32.21 mg/dL). Normalization of bilirubin concentration was achieved in 51 (58%) children from the study group before discharge. In the study group, the concentration of total bilirubin in cord blood was higher (4.26 mg/dL ± 1.8 vs. 2.39 mg/dL ± 1.4; *p* < 0.001) as well as the highest concentration of total bilirubin determined during hospitalization (15.27 mg/dL ± 5.8 vs. 10.24 mg/dL ± 3.4; *p* < 0.001). We also found lower hemoglobin levels at birth in the cholestasis group (14.01 g/dL ± 3.8 vs. 16.39 ± 2.8, *p* < 0.001) and lower hemoglobin levels during the entire hospitalization (nadir hemoglobin levels 10.84 g/dL ±3.5 vs. 15.57 g/dL ± 3.0, *p* < 0.001). Ferritin concentration was measured in 64.4% of children with cholestasis and only 29% of controls. No difference was observed in ferritin concentration between the groups. The results are presented in Table 2.

The number of children requiring top-up transfusions during hospitalization in the neonatal unit differed significantly: 33% of children with cholestasis and 3.8% of children with normal direct bilirubin levels needed a transfusion (*p* < 0.001). A comparison of the therapeutic procedures performed in the study and control groups revealed that children with cholestasis required phototherapy more often and for more extended periods. Similarly, they more often were administered immunoglobulin infusion (55.7% vs. 8.1%; *p* < 0.001), required exchange transfusion (8% vs. 2.2%; *p* = 0.042), as well as intravenous infusions of 10% glucose or parenteral nutrition (45.5% vs. 12.9%; *p* < 0.001), with withholding of enteral nutrition (8% vs. 2.2%; *p* = 0.042) and antibiotic therapy (14.8% vs. 4.8%; *p* = 0.005). The overall length of stay was also longer in this group (13.02 ± 9.3 days vs. 6.19 ± 4.3 days; *p* < 0.001).

In our cohort, no other significant abnormalities accompanied severe cholestasis. Mild abnormalities (such as stool color changes, hepatosplenomegaly, and low vitamin D concentration) were observed only in the cases of two children with extreme direct bilirubin levels. No coagulation disorders were found. Ferritin concentration was measured in 64.4% of children with cholestasis and only 29% of controls. No difference was observed in ferritin concentration between the groups. The results are presented in Table 3.

## 4. Discussion

Our study revealed that the incidence of cholestasis, defined as a bilirubin level exceeding 1 mg/dL, is 32.1% in newborns with HDFN. The significant risk factors for cholestasis were lower gestational age at birth, Rh or Kidd alloimmunization, and a history of intrauterine transfusions. The study is one of only a few that concentrate on this topic, and the first to be performed in a Polish cohort. The strength of the study is that it includes a relatively numerous group. A recently published Swedish study identified 149 newborns with HDFN delivered in the years 2006–2015 in the region of Stockholm, with a 7% incidence of cholestasis (defined as direct bilirubin level > 2 md/dL) [20]. Another study from Leiden published in 2011 identified 313 newborns with HDFN, of which 41 had cholestasis. They used, however, a different definition of cholestasis: direct bilirubin concentration above 1 mg/dL if total bilirubin below was 5 mg/dL, or direct bilirubin concentration exceeding 20% of the total if total bilirubin was higher than 5 mg/dL [4].

Although HDFN is rarely mentioned as a cholestasis-causing disorder, elevated direct bilirubin levels are noted in approximately 13% of cases [4,5,21]. The definition varies, however, among the studies. Some authors defined cholestasis when direct bilirubin concentration exceeded 2 mg/dL; other criteria were adopted in the Leiden study as mentioned above [4,20]. We decided to set the diagnostic threshold at lower bilirubin concentrations based on the guidelines published in 2017 [1,2,3,21,22]. The difference in the diagnostic approach influenced the results, and in our material, more than one-third of subjects (88/274; 32.1%) diagnosed with HDFN had elevated (>1 mg/dL) direct bilirubin levels. Additionally, a selection bias towards more severe HDFN cases with more frequent cholestasis treated in our unit may emerge from the fact that it was a reference center for fetomaternal immunization at that time. 

According to the literature, a higher risk of cholestasis is found in children from pregnancies complicated by the Rh type of HDFN, who require intrauterine transfusions [4,5,20]. In our study, the incidence of cholestasis was higher in children whose hemolytic disease was caused by the presence of anti-D and Kidd antibodies and with a history of intrauterine transfusions, with no effect of time interval since the last IUT. Still, the children delivered after a shorter time since the last IUT more often suffered from severe cholestasis.

It has been described that hypoxia due to anemia and increased hematopoiesis in the liver may cause liver dysfunction in children with HDFN and that a higher risk of cholestasis is supposed to correlate with lower hemoglobin concentration at birth [4]. Our study confirmed this, finding lower hemoglobin levels at birth and a lower nadir of hemoglobin in the cases with cholestasis. 

According to the literature, low birth weight is also a factor in the incidence of cholestasis [4,20]. In our study, however, there was no correlation between birth weight and the incidence of cholestasis, nor was it diagnosed more often in LBW or hypotrophic babies. Nevertheless, we observed an association between cholestasis and lower gestational age at birth, which confirms observations in other studies [4,20].

It has also been described that cholestasis is more common in children with high total bilirubin levels at birth [4], which is confirmed in our study results. 

It is reported that hepatic dysfunction due to iron overload resulting from erythrocyte hemolysis might cause cholestasis in infants with HDFN [4,6,7,23,24]. In our cohort, however, there was no significant difference in ferritin concentrations between the groups. The limiting factor might be that ferritin concentration was not checked in all children in the study groups, but only in 64.4% of children in the group of children with cholestasis and 29% in the group of children without cholestasis. Moreover, due to the limitations of laboratory methods, the highest ferritin concentration (above 1650 ng/mL) was not determined as a quantifiable value, so the average ferritin concentration is only determined in children whose concentration was <1650 ng/mL. 

Demircioglu F. et al. described a case of a baby born at 33 weeks’ gestation complicated by Rh-type HDFN after two complementary intrauterine transfusions. The direct bilirubin level was 17.9 mg/dL, and the ferritin level was 8842 ng/mL, and a liver biopsy showed cholestasis and iron overload [23]. The author of this article has published a case report of a newborn with HDFN that had a maximum of 33.14 mg/dL total bilirubin and ferritin level above 33,000 ng/mL and was treated with chelation therapy [7].

The literature describes clinical problems experienced by patients born from pregnancies complicated by HDFN. Until recently, no cases of patients with a direct bilirubin concentration > 50 mg/dL have been reported.

Zonneveld R. et al. presented a case report of a patient born from a pregnancy complicated by an A/O conflict. The baby was born at term with anemia, jaundice, and moderate hepatomegaly. The maximum direct bilirubin concentration was 192 µmol/L (i.e., approximately 11.2 mg/dL), and the maximum ferritin concentration was 1138 pmol/L [25]. Grobler et al. described a case of a term newborn with Rh hemolytic disease-induced kernicterus, in whom the maximum concentration of direct bilirubin was 31.6 mg/dL and total bilirubin was 45.2 mg/dL [26]. Watchko et al., in their review, have included a case of a newborn with ABO hemolytic disease-induced kernicterus. The maximum concentration of the child’s total bilirubin was 61 mg/dL, and direct bilirubin was 27.7 mg/dL [27]. In our study, twins born at 34 weeks of gestation with a history of two IUTs because of anti-D and C immunization had maximum direct bilirubin levels of 50.2 mg/dL and 32.21 mg/dL, respectively. The detailed clinical course was reported previously [6].

In general, treatment of intrahepatic cholestasis depends on the etiology. Most cases resolve spontaneously on conservative treatment. Ursodeoxycholic acid, fat-soluble vitamins, DHA, and fish oil for parenteral nutrition and MCT mixture were used in several described patients. In some cases, iron chelates are used for treatment [4,7,23,24,28,29,30]. 

According to the literature, bilirubin concentrations in children with HDFN usually normalize spontaneously within one week to three months. It is recommended that patients be monitored until biochemical parameters normalize [4,20]. In the described group, the direct bilirubin concentration decreased in most cases during hospitalization—before discharge, normalization was observed in 51 (58%) patients.

In summary, the factors that contributed to the higher risk of cholestasis in children with pregnancies complicated by serologic conflict were lower gestational age, Rh or Kidd serological type of HDFN, and intrauterine transfusion. Children diagnosed with cholestasis had higher cord blood bilirubin levels, higher maximum total bilirubin levels, lower hemoglobin levels at birth, higher rates of postnatal blood transfusion, more frequent need for exchange transfusion, more extended time and higher rate of phototherapy, higher rate of immunoglobulin and antibiotic therapy, as well as more frequent need of parenteral nutrition. In the group of children with cholestasis, there were more often newborns in whom enteral feeding had to be postponed or withheld. In these children, abnormalities of the liver ultrasonography were found more often, and they required prolonged hospital stays. Interestingly, there was no difference in body weight, incidence of low birth weight, or fetal growth restriction between children with cholestasis and controls.

The results of this analysis should be interpreted with caution due to incomplete diagnostics in a significant proportion of newborns and the short follow-up period of these children. This study had several limitations. The main concern was missing data from the patients’ medical histories due to multicenter management, such as the number of top-up transfusions, and the retrospective design of the study.

## 5. Conclusions

After analyzing the medical history of patients born in our center in recent years, it seems that cholestasis may occur in more than 30% of patients with HDFN. The risk factors for cholestasis in children with HDFN are lower gestational age at delivery, Rh and Kidd serological type of HDFN, and the need for intrauterine transfusions. Direct bilirubin concentrations in selected cases may exceed the norm several dozen times. Further studies with planned extended diagnostics and at least several years of follow-up are needed.

## Figures and Tables

**Table 1:** Study and control group characteristics (*p* < 0.05).

	Newborns		
	With Cholestasis (*n* = 88)	Without Cholestasis (*n* = 186)	*p*
Females (%)	36 (40.9)	92 (49.5)	0.310
Males (%)	52 (59.1)	94 (50.5)	0.310
Weeks at delivery	36.83 ± 1.9	37.57 ± 1.8	0.002
Prematurely born newborns (%)	36 (40.9)	48 (25.8)	0.011
Birthweight	3125.19 ± 548.2	3175.60 ± 485.8	0.443
No. of LBW newborns (%)	9 (10.2)	14 (7.5)	0.452
Birthweight percentile (%)	6638 ± 25.1	61.69 ± 25.3	0.152
No. of SGA newborns (%)	1 (1.1)	2 (1.1)	1.000
Serological type of HDFN (%)			
Rh	66 (75.0)	98 (52.7)	<0.001
Kidd	5 (5.7)	1 (0.5)	0.014
Kell	3 (3.4)	6 (3.2)	1.000
Duffy	2 (2.3)	5 (2.7)	1.000
MNs	1 (1.1)	2 (1.1)	1.000
ABO	19 (21.6)	80 (43.0)	0.001
Several antigens	7 (8.0)	6 (3.2)	0.125
No. of fetuses requiring IUT (%)	24 (27.3)	22 (11.8)	0001
No. of IUT in IUT-treated neonates	2.63 ± 1.8	4.64 ± 2.9	0.008
Time since last IUT [days]	15.83 ± 7.3	13.95 ± 4.7	0.301

**Table 2:** Selected laboratory parameters in the study and control groups.

	Newborns		
	With Cholestasis (*n* = 88)	Without Cholestasis (*n* = 186)	*p*
DB–first concentration [mg/dL]	0.63 ± 0.6	-	-
DB–max concentration [mg/dL]	2.56 ± 6.2	-	-
Highest DB concentration–days of life	5.99 ± 3.8 5.00 (4.00–7.00)	-	-
DB concentration at discharge [mg/dL]	1.08 ± 0.9	-	-
No. of newborns with discharge DB concentration of at least 1.0 (%)	37 (420)	-	-
Hgb concentration at birth [g/dL]	14.01 ± 3.8	16.39 ± 2.8	<0.001
Lowest Hgb concentration [g/dL]	10.84 ± 3.5	15.57 ± 3.0	<0.001
TB concentration in umbilical cord blood [mg/dL]	4.26 ± 1.8	2.39 ± 1.4	<0.001
No. of newborns with TB concentration measured in umbilical cord blood (%)	82 (93.2)	149 (80.1)	
Highest TB concentration [mg/dL]	15.27 ± 5.8	10.24 ± 3.4	<0.001
Highest TB concentration–days of life	5.48 ± 3.0	4.18 ± 1.7	<0.001
No. of newborns with ferritin concentration measured (%)	56 (64.4)	54 (290)	-
Mean ferritin concentration [mg/dL] ^1^	835.71 ± 3714	678.78 ± 479.2	0.058
No. of newborns with ferritin concentration above 1650 mg/dL ^2^ (%)	3 (34)	5 (2.7)	0.714

DB—direct bilirubin; TB—total bilirubin; IUT—intrauterine transfusion; *p* < 0.05. ^1^ Only ferritin concentration < 1650 ng/mL. ^2^ Due to laboratory limitations, higher concentrations were labeled as above the test’s detection range (>1650 ng/mL).

**Table 3:** Clinical management and diagnostic tests in the study and control groups.

	Newborns		
	With Cholestasis (*n* = 88)	Without Cholestasis (*n* = 186)	*p*
No. of newborns requiring blood transfusions (%)	29 (33.0)	7 (3.8)	<0.001
No. of TUs in TU-treated neonates	1.4 ± 0.6	1.14 ± 0.4	0.217
No. of newborns requiring exchange transfusions (%)	7 (8.0)	4 (2.2)	0.042
No. of ETs in ET-treated neonates	1.14 ± 0.4	1.00 ± 0.0	0.479
No. of newborns requiring phototherapy (%)	83 (94.3)	110 (59.1)	<0.001
Duration of phototherapy in phototherapy-treated neonates [days]	8.78 ± 3.8	4.07 ± 2.5	<0.001
No. of newborns who received immunoglobulin infusions (%)	49 (55.7)	15 (8.1)	<0.001
No. IVIG infusions in IVIG-treated neonates	1.57 ± 0.8	1.27 ± 0.6	0.123
No. of newborns requiring TPN/PPN/IV fluids (%)	40 (45.5)	24 (12.9)	<0.001
Duration of TPN/PPN/IV fluids in neonates with PN [days]	4.88 ± 4.9	3.58 ± 4.4	0.295
No. of newborns who received ATB (%)	13 (14.8)	9 (4.8)	0.005
Duration of ATB in ATB-treated neonates [days]	7.15 ± 4.7	5.33 ± 4.1	0.355
No. of children with postponed or suspended oral feeding (%)	7 (8.0)	4 (2.2)	0.042
Days with postponed or suspended oral feeding Among those who required postponed or suspended oral feeding	4.00 ± 4.3	5.25 ± 6.0	0.694
No. of newborns who had abdominal USG performed (%)	67 (76.1)	63 (33.9)	-
Abnormal findings in abdominal USG (%)	12 (17.9)	4 (6.3)	0.045
Duration of hospitalization [days]	13.02 ± 9.3	6.19 ± 4.3	<0.001
No. of deceased newborns (%)	2 (2.3)	1 (0.5)	0.242
No. of newborns presenting with acholic stools (%)	2 (2.3)	0 (0.0)	-

DB—direct bilirubin; TB—total bilirubin; IUT—intrauterine transfusion; TPN/PPN—total/peripheral parenteral nutrition; ATB—antibiotics; *p* < 0.05.

## Data Availability

Data presented in this study are available in Table 1, Table 2 and Table 3. Additional data available upon request.
